# Differential role of r-met-hu G-CSF on male reproductive function and development in prepubertal domestic mammals

**DOI:** 10.1371/journal.pone.0222871

**Published:** 2019-09-26

**Authors:** Pedro M. Aponte, Miguel A. Gutierrez-Reinoso, Edison G. Sanchez-Cepeda, Manuel Garcia-Herreros

**Affiliations:** 1 Colegio de Ciencias Biológicas y Ambientales, Universidad San Francisco de Quito (USFQ), Campus Cumbayá, Quito, Ecuador; 2 Colegio de Ciencias de la Salud, Escuela de Medicina Veterinaria, Universidad San Francisco de Quito (USFQ), Campus Cumbayá, Quito, Ecuador; 3 Instituto de Investigaciones en Biomedicina “One-health”, Universidad San Francisco de Quito (USFQ), Campus Cumbayá, Quito, Ecuador; 4 Facultad de Ciencias Agropecuarias y Recursos Naturales, Carrera de Medicina Veterinaria, Universidad Técnica de Cotopaxi (UTC), Latacunga, Ecuador; 5 AGROCALIDAD, Ambato, Ecuador; 6 National Secretariat *of* Higher Education, Science, Technology and Innovation (SENESCYT), Quito, Ecuador; 7 Instituto Nacional de Investigação Agrária e Veterinária, I. P. (INIAV, I.P.), Polo de Santarém, Santarém, Portugal; Zhejiang University College of Life Sciences, CHINA

## Abstract

The understanding of mammalian spermatogenesis niche factors active during sexual development may be leveraged to impact reproduction in farm animals. The aim of this study was to evaluate the effects of r-met-hu/G-CSF (filgrastim) on prepubertal sexual development of guinea pigs (*Cavia porcellus*) and ram lambs (*Ovis aries*). Individuals of both species were administered r-met-hu/G-CSF daily for 4 days. During and after administration protocols, testicular function and development were assessed through hematological responses, hormonal profiles (gonadotropins, testosterone and cortisol) testicular morphometry and germ cell kinetics. As expected, r-met-hu/G-CSF acutely mobilized white-lineage blood cells in both species. LH was increased by r-met-hu/G-CSF in guinea pigs (P<0.01) but T remained unchanged. In ram lambs gonadotropins and T increased in dose-response fashion (P<0.01) while cortisol values were stable and similar in treated and control animals (P>0.05). In guinea pigs there were no differences in testicular weights and volumes 2-mo after r-met-hu/G-CSF application (P>0.05). However, ram lambs showed a dose-response effect regarding testis weight (P<0.05). 66.66% of ram lambs had initial testes not yet in meiosis or starting the first spermatogenic wave. After 60-days only 25% of control animals were pubertal while all treated animals (1140-μg) had reached puberty. We propose an integrated hypothesis that G-CSF can stimulate spermatogenesis through two possible ways. 1) r-met-hu/G-CSF may go through the brain blood barrier and once there it can stimulate GnRH-neurons to release GnRH with the subsequent release of gonadotrophins. 2) a local testicular effect through stimulation of steroidogenesis that enhances spermiogenesis via testosterone production and a direct stimulation over spermatogonial stem cells self-renewal. In conclusion, this study shows that r-met-hu/G-CSF differentially affects prepubertal sexual development in hystricomorpha and ovine species, a relevant fact to consider when designing methods to hasten sexual developmental in mammalian species.

## Introduction

Spermatogenesis is a complex physiologic process in which spermatogonial cells differentiate into spermatozoa after mitosis, meiosis, and spermiogenesis [1]. In mammals, spermatogenesis is maintained by spermatogonial stem cells (SSCs) which depend on Sertoli cells for proliferation, differentiation, and survival [1–3]. SSCs reside in a stem cell niche that constantly regulates the steady state of spermatogenesis. Efficient sperm production will be the result of an accurate cross-talk of SSCs with Sertoli cells and other testicular niche cells involving growth factors and cytokines [3,4].

Starting the prepubertal period the germinal epithelium initiates spermatogenesis for the first time under the influence of several hormone pulses (e.g. LH, FSH) and when sperm cells appear in the seminiferous tubules, male animals reach puberty [1,5,6]. The first step of this spermatogenic wave requires gonocytes to transform into SSCs at the basal compartment of the seminiferous tubules [1,2]. After several rounds of mitosis, spermatocytes are formed that undergo genetic recombination (meiosis) to yield round spermatids, the direct precursors of sperm [1–3]. Thus, the onset of spermatogenesis is characterized by an increase in germ cell (GCs) numbers [1–4,6], representing a gradual and complex activation of sets of differentiation genes. The study of the prepubertal period and the role of different spermatogenic factors involved is important for the prediction of future reproductive performance in adult males, with the goal of better impacting the reproductive management of farm animals [7,8].

Granulocyte colony-stimulating factor (G-CSF) is a member of the hematopoietic growth factor family [9–11]. G-CSF is a cytokine (175 aa; MW = 18.8 kDa) with several physiological roles including the stimulation of hematopoietic cell proliferation, differentiation and maturation [12,13]. However, there is evidence supporting new physiological roles for G-CSF in other non-hemopoietic organs [12–14]. G-CSF is produced by Leydig and Sertoli cells in the testis and is present in the seminal plasma [14–18]. Moreover, G-CSF receptor CSF3R has been identified in murine SSCs [19]. Testicular damage from chemotherapy influence the physiological regeneration of male GCs in the testis [20,21], up to a limit imposed by Sertoli cell numbers [20] and G-CSF seems to have a protective role in radiation-induced testicular impairment [21] Currently, there are several types of human recombinant G-CSF (rhG-CSF) commercially available for clinical applications [22–24]. The non-glycosylated r-met-hu G-CSF, known as r-met-hu/G-CSF, has been successfully used to stimulate proliferation and maturation of hematopoietic cells [11,25]. However, the physiological role of exogenous r-met-hu G-CSF on non-hematopoietic tissues such as testicular tissue and GCs proliferation and differentiation is still unknown. The possibility of using r-met-hu G-CSF for the stimulation of sexual maturity in domestic animals would open new venues with potential for higher profits in the animal production field [26–28]. Early puberty induction would make superior genetic material available ahead of time, representing interesting economical and genetic implications [26,28] like extended life-times that would reduce overall operational costs and lower the intergenerational intervals, thus, maximising the genetic improvement and business profitability. Additional advantages include the fact that younger sires are easier to manage, faster learners, have superior fitness and importantly, a higher libido compared to older sires [29].

In order to gain insight into the possible roles of r-met-hu G-CSF on male sexual function and development during the early prepubertal period, the aim of the present study was to evaluate its effects by monitoring hematological responses, endocrine profiles, testicular tissue development and germ cell kinetics in a prepubertal laboratory animal, guinea pig (*Cavia porcellus*) and a farm animal ram lambs (*Ovis aries*).

## Materials and methods

### Ethical statement

This research was performed under the Project License PROM-CEB-013-2014 from the National Secretariat of Higher Education, Science, Technology, and Innovation (SENESCYT), in strict accordance with the recommendations in the legal framework (Animal Welfare Law) currently in place in AGROCALIDAD Ethics Committee (MAGAP- AGROCALIDAD), for all Ecuadorian Public and Private Laboratories and Higher Education Institutions (Ecuadorian Animal Protection Law; COIP Art. 249–2014). "This research was performed under the Project License PROM-CEB-013-2014 from the National Secretariat of Higher Education, Science, Technology, and Innovation (SENESCYT), in strict accordance with the recommendations in the legal framework (Animal Welfare Law) currently in place and approved by AGROCALIDAD Ethics Committee (MAGAP- AGROCALIDAD), and under regulations for all Ecuadorian Public and Private Laboratories and Higher Education Institutions (Ecuadorian Animal Protection Law; COIP Art. 249–2014). Guinea pigs were castrated under general anesthesia (ketamine + xylacine). For castration, ram lambs were sedated with xylacine and local anesthesia was used (lidocaine). Analgesics were used for 24h after surgery (Guinea pigs: aspirin; ram lambs: flunixin)"

### Reagents and media

All reagents and media used in the present study were purchased from Sigma (Sigma-Aldrich, St Louis, MO) unless otherwise stated. In all cases, reagents and media were used according to manufacturer recommendations.

### Animals and management

Entire male guinea pig males (*Cavia porcellus;* n = 32) from Peruvian genetic-line (age 1-mo) were used for the different experiments. All animals were randomly sorted by experimental groups and kept in indoor individual boxes (0.6×0.75×0.5 m). Entire Corriedale breed ram lambs (*Ovis aries*; n = 37; age 2-mo) were maintained in indoor individual pens (5×5 m). The buildings for the housing of the animals were disinfected with quaternary ammonium (day -7) every 7d. In all cases, vitamins and dextrose were given to attenuate transportation and initial stress. All animals were quarantined for 15d before the experiments started to ensure a disease-free status and adaptation to their new environment. During adaptation (8-days) all animals were dewormed, kept in an enriched environment and fed alfalfa forage, standard formulated concentrate-food and water *ad libitum* during the experimental period.

### r-met-hu G-CSF treatment outline

Irrespective of the species studied, individuals were randomly allocated to experimental groups. In a first experiment, 5 control and 15 treatment animals were used. In both species each treatment consisted in 4 daily SC applications of r-met-hu G-CSF (Filgen^®^, Bioprofarma, Buenos Aires, Argentina). In guinea pigs, three r-met-hu G-CSF treatment groups (n = 5, each) were administered 1 μg, 10 μg and 20 μg respectively per animal for 4 days (total in 4 days = 4, 40 and 80 μg, respectively). In ram lambs, three r-met-hu G-CSF treatment groups (n = 5 each) received 18 μg, 180 μg and 360 μg per animal respectively, daily for 4 days (total in 4 days = 72, 720 and 1440 μg, respectively). The control group (n = 5) was administered with saline (NaCl 0.9% w/v), instead of r-met-hu G-CSF. In order to evaluate r-met-hu G-CSF treatment effect on testicular morphometrics all animals were castrated (final age = three and four months for guinea pigs and ram lambs, respectively). After assessing the results, 12 or 17 animals (guinea pigs or ram lambs, respectively) of the same ages, species, husbandry and sources were used for a second experiment to evaluate spermatogenesis through histological and stereological methods. On this second experiment, 4 or 6 initial control animals were castrated at day-0 (age 1 mo for guinea pigs and 2 mo for ram lambs, respectively), 4 guinea pigs were treated with the highest dose (as stated for experiment 1, ram lambs n = 5) and 4 were controls (ram lambs n = 6). r-met-hu G-CSF treated animals and controls were castrated at ages 3 mo (guinea pigs) and 4 mo (ram lambs). Table 1 summarizes the number of animals used in the experimental set-up of this study.

**Table 1:** Summary of animals used.

Group	Experiment 1 (n) *C*. *porcellus*	Experiment 1 (n) *O*. *aries*	Experiment 2 (n) *C*. *porcellus*	Experiment 2 (n) *O*. *aries*
**Initial control**	---	---	4	6
**Control**	5	5	4	6
**Treatment 1**	5	5	---	---
**Treatment 2**	5	5	---	---
**Treatment 3**	5	5	4	5
**TOTAL**	20	20	12	17

Initial control corresponds to animals at the beginning of the experiments used to evaluate initial developmental status of the testis. Control included animals that were administered saline (NaCl 0.9%) daily for 4 days. Treatments consisted on injecting r-met-hu/G-CSF daily to animals for 4 days. Treatment 1 is the lowest dose (guinea pigs 1 μg/animal/day; ram lambs 18 μg/animal/day). Treatment 2 is the intermediate dose (guinea pigs 10 μg/animal/day; ram lambs 180 μg/animal/day). Treatment 3 is the highest dose (guinea pigs 20 μg/animal/day; ram lambs 360 μg/animal/day).

### Hematological assessment

Hematologic analysis (hemograms) provided essential information to monitor bone marrow response to different r-met-hu G-CSF treatments. Changes in cell profiles were evaluated at days 1,2,3,4 and 5 post-treatment. Hemograms were carried out with a HumaCount 30TS® hematologic analysis system (Human GmbH, Wiesbaden, Germany). The analysis included all available red blood cell (RBC) and differential white blood cell (WBC) counts and indices. RBC count and indices included mean corpuscular volume (MCV) and red cell distribution width (RDW) among others. Differential WBC counts included total WBC count, absolute granulocyte count (AGC), absolute lymphocyte count (ALC), absolute monocyte count (AMC). Wright stained smears were evaluated by using a Human Scope® microscope (Human GmbH, Wiesbaden, Germany) to assess relative values.

### Endocrine profile assays

Blood samples (1 mL) from guinea pigs were aseptically taken for hormone analysis (LH, FSH, T and cortisol) by making a small incision at the interdigital space, once, 5 days after the first r-met-hu/G-CSF injection. In ram lambs, 5 mL-blood samples were obtained from the external jugular vein. For each individual, a blood sample was taken at days 1, 5, 15, 30 and 60 (total blood samples = 5). A blood sample was taken to the group of animals castrated at the beginning of the experiments (before castration). Hormonal assays were performed at a private accredited bioanalysis laboratory. Hormonal assays were performed using the enzyme-linked immunosorbent assay (ELISA) kits (Abnova, Taipei City, Taiwan). For the direct quantitative determination of FSH, LH, T and Cortisol, specific monoclonal antibodies (anti-FSH-RP, anti-LH-RP, anti-Testosterone-RP and Anti-Cortisol-RP, respectively) were used. A two-step assay was carried out for the quantification of each hormone. The first monoclonal specific antibodies were used as first immobilized factors onto the microwell plate and the second specific antibodies, conjugated with radish peroxidase (RP), were designed for a different specific region of each hormone.

### Testicular tissue sampling and processing

Sixty days after the last r-met-hu G-CSF application all animals were castrated (control and treatment groups). Guinea pigs were castrated in a surgical area, under general anesthesia (ketamine, 40 mg/kg/IP; xylacine 5mg/kg/IP). For castration, ram lambs were sedated with xylacine (0.1mg/kg/IV) and local anesthesia was used (lidocaine) in a clean environment (barn). Guinea pigs were administered aspirin (85 mg/Kg orally, every 4 hours for 24 hours). Ram lambs were given flunixin (1 mg/Kg, SC) immediately after the surgery and a second dose after 24h. Animals were monitored and showed no signs of pain or abnormal behavior during the first 72h after surgery (guinea pigs were also checked during the first night after surgery). Testes were obtained early in the morning and kept at ~4 ^o^C before histological fixation. The *tunica vaginalis* and epididymis were excised and testis weight recorded. Volume was estimated by the water displacement method using a graduated cylinder. Testes did not show any apparent macroscopical abnormalities. Testes were transversally excised and tissue samples were fixed in neutral buffered formalin (4%v/v formaldehyde, 48h) or Bouin’s fixative and stored in 70%v/v ethanol (4 ^o^C). Testicular tissue pieces (~0.5 cm^3^) were transferred into biopsy cassettes for further histological processing. Samples were dehydrated in alcohol series and xylene and infiltrated with paraffin in a histoprocessor (Slee MTM, Mainz, Germany). Samples were embedded in paraffin blocks and cut 5-μm thick with a microtome (Cut 6062, Slee, Mainz, Germany). Slides were deparaffinised, stained with Harris hematoxylin and mounted with Secure Mount^tm^, (Fisherbrand, Pittsburgh, PA, USA).

### Testicular morphometry and histological state assessment

Testicular tissues were analyzed for general morphology. Stereologic and morphometric parameters were estimated. Images were captured with a digital video camera (Tucsen Imaging Technology Co., Ltd, Beijing, China). Tubule diameters were measured on the digital images. Tubular diameters were calculated as the average of the major and minor axes of randomly chosen near-round cross-section profiles of seminiferous tubules. Diameters were measured with image-J software (downloaded from http://imagej.nih.gov/ij/) in an average of 80 and 200 cross-sections of tubule profiles/animal (guinea pigs and ram lambs respectively).

### Determination of testicular developmental status

Phases of testicular development considered were neonatal, spermatogonial expansion, meiotic, spermiogenesis and pubertal. An animal was considered to match a developmental period if the specific most-advanced GC type was present in more than 10% of seminiferous tubules cross-sections. At least 60 seminiferous tubule cross-sections per animal were observed and recorded to this aim. During the neonatal period the predominant GC type are gonocytes. Spermatogonial phase implies that the first round of spermatogenesis has already begun and the most advanced GC type are spermatogonia (types A, In or B). During the meiotic phase animals show primary spermatocytes as the most advanced GC types. Animals were considered in spermiogenesis when spermatids (in any step) were the predominant GC type. Pubertal animals were those with at least 10% of seminiferous tubule cross-sections with sperm in the lumen.

Daily sperm production (DSP) and efficiency of spermatogenesis (DSP/g of testicular parenchyma) were estimated and compared among treatments in animals with full spermatogenesis. To this end, digital images of 20 random microscopic fields at 400x were captured and used for round spermatid counting through the physical dissector [30]. Each image had a look-up image exactly registered from an immediate consecutive section obtained during microtome sectioning. Unbiased estimated spermatids counts were used to feed the formula: DSP = (total spermatid number per testis x theoretical number of sperm produced by each spermatid) / time divisor. Total numbers of spermatids per testis were estimated by first obtaining spermatid density (spermatids/mL) through 16 stereological volume probes (*Cavia porcellus*: 12500 μm^3^ each, total of 200000 μm^3^ per image; *Ovis aries*: 200000 μm^3^ each, total of 3200000 μm^3^ per image) and by multiplying this density by the testicular volume. Clearly, each spermatid produces one sperm while the time divisor (which is considered a constant for a species) is the sum of the duration of the stages (in days) were round spermatids appear. To this end, the duration of the cycle and frequency of the stages is required. For guinea pigs, the time divisor is 2.57 (sum of the duration in days of stages I through IV, that is, 0.93, 0.56, 0.33 and 0.76, calculated through the frequencies of the respective stages, 11%, 6.58%, 3.91% and 8.94%, respectively) in reference to the total duration of the cycle (8.45 days) [31]. For ram lambs, the time divisor was 7.18 (sum of the duration in days of stages I and V through VIII, that is, 2.6, 0.7, 1.7, 0.8 and 1.3, calculated through the frequencies of those stages, 25.4%, 6.6%, 16.7%, 8.1% and 12.2%, respectively) in reference to the total duration of the cycle (10.4 days) [32].

### Statistical analysis

As the effect of r-met-hu G-CSF on the reproductive status of farm and lab animals has not been widely studied and there are not enough data available (particularly statistical distributions and variance information), power calculation for animal number estimation was not performed previous to the start of our experiments. Numbers of animals used for the treatments used are specified in the figure legends. Every animal participating on this study was considered an experimental unit. All statistical analyses were performed using SPSS for Windows® software, v17. General statistical analysis of haematological values, hormonal levels, stereologic and morphometric variables was performed using one-way ANOVA. The level of significance was set at P<0.05 and when the analyses were statistically significant, multiple comparisons were performed by means of Bonferroni *post-hoc* test.

## Results

### Effects of r-met-hu G-CSF treatment on hematological parameters

**Fig 1:**
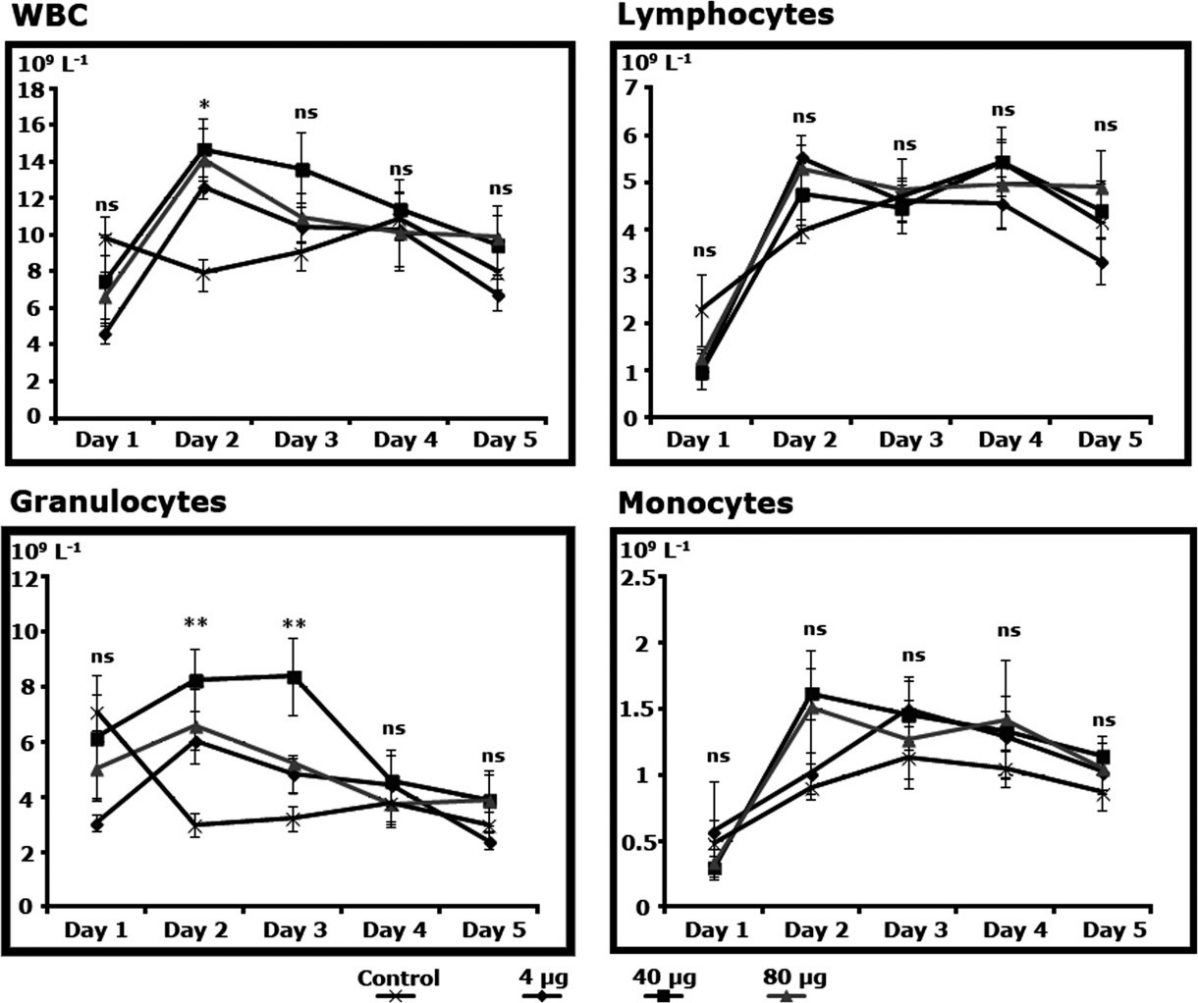
Hematodynamics of r-met-hu G-CSF in guinea pigs (*Cavia porcellus*). Differences, when detected, refer to treatments on the same day. (*) P<0.05; (**) P<0.01; (ns) non significant. Control n = 5; dose 4μg n = 5; dose 40μg n = 5; dose 80μg n = 5.

In r-met-hu G-CSF treated guinea pigs white blood cells (WBCs) increased in numbers between days 1 and 2, irrespective of the doses used compared to the control (P<0.05) to later on decrease to control levels. WBCs response was mostly based in an increase in granulocytes which had a sustained response compared to the controls until day 3 (P<0.01), when levels dropped back to control values (Fig 1). Red blood cells (RBCs) did not show differences among treatments (P>0.05).

**Fig 2:**
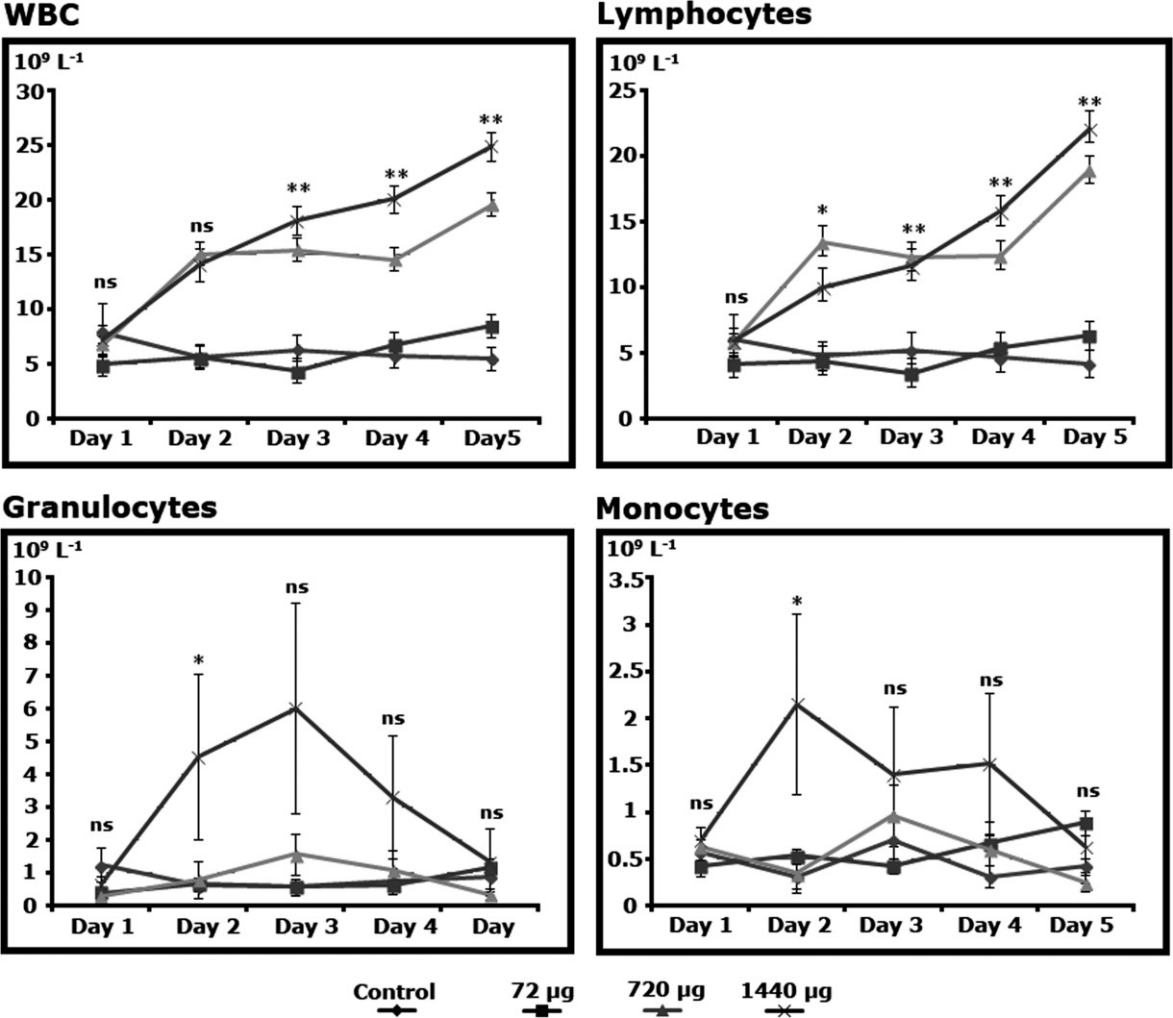
Hematodynamics of r-met-hu G-CSF in ram lambs (*Ovis aries*). Differences, when detected, refer to treatments on the same day. (*) P<0.05; (**) P<0.01; (ns) non significant. Control n = 5; dose 72μg n = 5; dose 720μg n = 5; dose 1440μg n = 5.

The patterns of bone marrow response after administering r-met-hu G-CSF differed in ovine species. WBCs in treated ram lambs had consistent higher values after day 3, significant with highest doses of r-met-hu G-CSF (720 μg and 1140 μg), (P<0.01), (Fig 2). This pattern was also followed by lymphocytes, but starting at day 2 (P>0.05) (Fig 2). Granulocytes and monocytes increased their numbers by day 2, only with the highest dose (1140 μg), (P<0.05), to later decrease to control and the lowest dose (72 μg) response levels (Fig 2).

### Effects of r-met-hu G-CSF application on hormonal profiles

**Fig 3:**
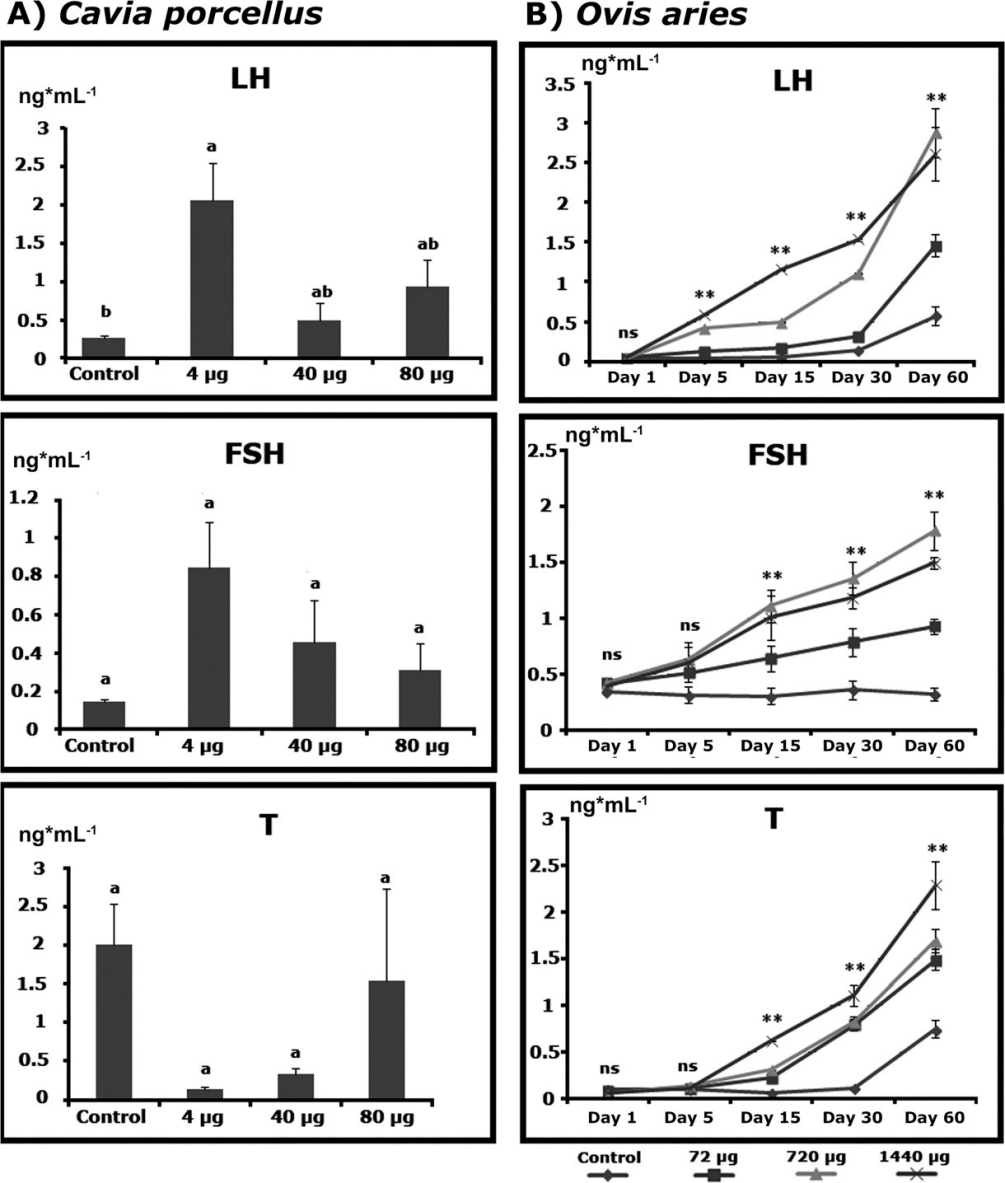
**Effect of r-met-hu G-CSF on hormonal levels: gonadotropins (FSH and LH) and Testosterone (T) in (A) guinea pigs (*Cavia porcellus*) at day 5 post- administration and (B) in ram lambs (*Ovis aries*) during 5 days post- administration.** In guinea pig experiments different superscripts represent significant differences. In ram lambs, differences, when detected, refer to treatments on the same day. (*) P<0.05; (**) P<0.01; (ns) non significant. *C*. *porcellus*, Control n = 5; dose 4μg n = 5; dose 40μg n = 5; dose 80μg n = 5; *O*. *aries*, Control n = 5; dose 72μg n = 5; dose 720μg n = 5; dose 1440μg n = 5.

Different hormonal patterns as responses to r-met-hu G-CSF were found in guinea pigs and ram lambs. In guinea pigs at day 5, r-met-hu G-CSF was able to significantly enhance LH secretion compared to the control group (P<0.01), an effect more clear with the lowest dose (4 μg), while FSH values showed a similar pattern, though statistically *ns*. Testosterone values were unchanged in treated guinea pigs with respect to controls (P>0.01), (Fig 3A).

In r-met-hu/G-CSF treated ram lambs, gonadotropins and testosterone values constantly and clearly increased during the first 5 days compared to the controls, in a doses–response fashion (P<0.01), (Fig 3B). At day five LH, FSH and testosterone were approximately 4.7, 4.0 and 2.5-fold higher respectively than the value of the control animals. Cortisol values in ram lambs did not change through the blood sample collection period (P>0.05), with a pooled value of 0.87±0.12 μg/dL.

### Effects of r-met-hu G-CSF application on testicular morphometric parameters

r-met-hu G-CSF had different sets of effects depending on the species studied. In guinea pigs there were no differences in treated animals compared the control group regarding testicular weight and volume 2-mo after the r-met-hu/G-CSF administration (P>0.05), (Table 2), while in ram lambs there was a clear doses-response effect in testis weight (P<0.05), (but not in testis volume, P>0.05), an effect significantly higher with the highest r-met-hu/G-CSF doses (P<0.01), (Table 2).

**Table 2:** Effect of r-met-hu G-CSF (2 mo. after administration) on testis weight and volume obtained after castration in guinea pigs (*Cavia porcellus;* 3 mo of age) and ram lambs (*Ovis aries;* 4 mo of age).

FILGRASTIM TREATMENT (FT) (Dose x days)	TESTES WEIGHT (TW; gr) (Right+Left Testis) (Mean±SEM)	TESTES VOLUME (TV; mL) (Right+Left Testis) (Mean±SEM)
	*Cavia porcellus* (Guinea pig)	
**FT1 (Control)**	6.73±0.30^a^	6.39±0.28^a^
**FT2 (1 μg x 4 d.)**	7.10±0.38^a^	6.75±0.36^a^
**FT3 (10 μg x 4 d.)**	5.55±0.29^a^	5.28±0.27^a^
**FT4 (20 μg x 4 d.)**	6.15±0.33^a^	5.86±0.30^a^
	*Ovis aries* (Ram lamb)	
**FT1 (Control)**	30.03±3.16^a^	10.27±1.37^a^
**FT2 (18 μg x 4 d.)**	37.02±3.00^ab^	30.38±1.45^b^
**FT3 (180 μg x 4 d.)**	40.60±1.16^bc^	43.10±1.48^b^
**FT4 (360 μg x 4 d.)**	42.01±2.00^bc^	38.16±1.63^b^

GSI = [gonad weight / total tissue weight] × 100. Different superscripts (a-c) represent significant differences (p≤ 0.05).

**Fig 4:**
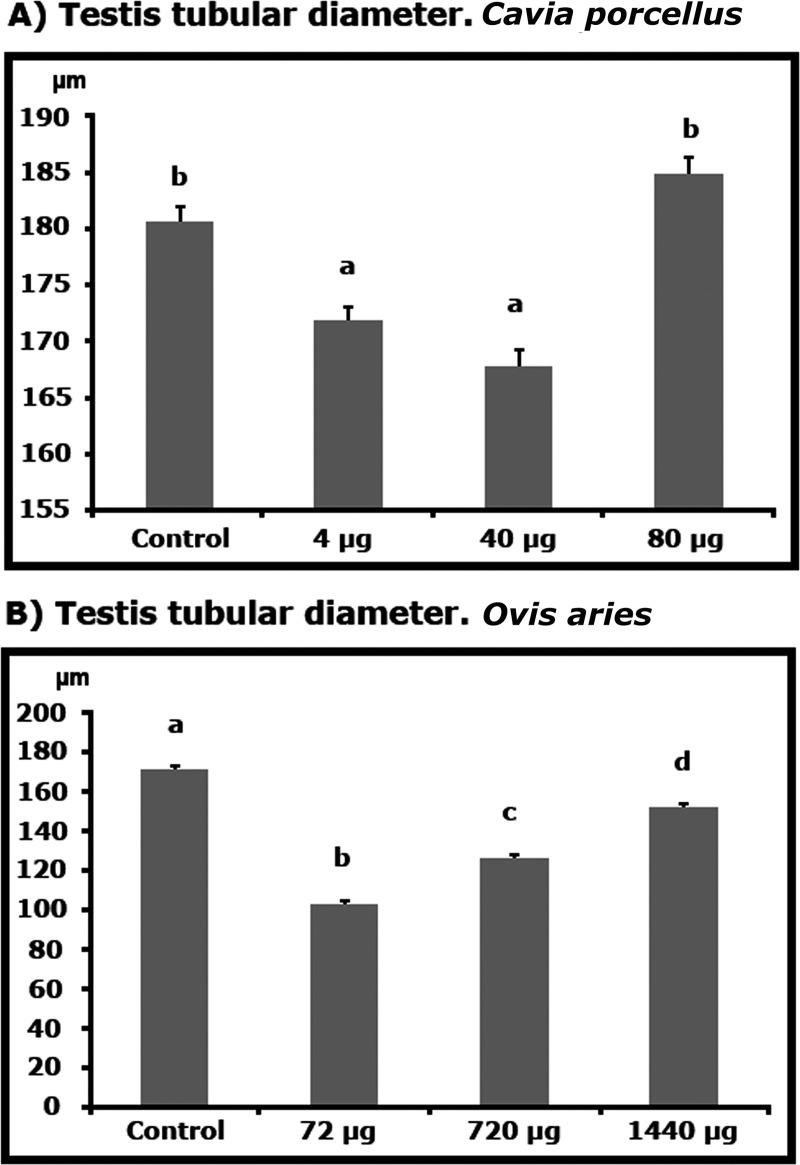
**Effect of r-met-hu G-CSF on the diameters of seminiferous tubule cross-sections in (A) guinea pigs (*Cavia porcellus*) obtained through morphometry from histological images after castration, 2 mo post- administration and in (B) ram lambs (*Ovis aries*) 2 mo post- administration.** Different superscripts (a-c) represent significant differences (P≤ 0.05). *C*. *porcellus*, Control n = 5; dose 4μg n = 5; dose 40μg n = 5; dose 80μg n = 5; *O*. *aries*, Control n = 5; dose 72μg n = 5; dose 720μg n = 5; dose 1440μg n = 5.

Seminiferous tubule diameters were significantly lower in guinea pigs treated with the lowest doses of r-met-hu/G-CSF compared to control animals (r-met-hu/G-CSF treated: diameter range = 165–175 μm; control: range = 180–185 μm; P<0.05). However, the diameters of animals treated with higher doses were similar to those of the control (Fig 4A). Ram lambs had smaller seminiferous tubule diameters with all r-met-hu/G-CSF doses than control animals However, seminiferous tubule cross-sections with the highest r-met-hu/G-CSF dose had the highest diameters (P<0.01), (Fig 4B).

### Germ cell kinetics

**Fig 5:**
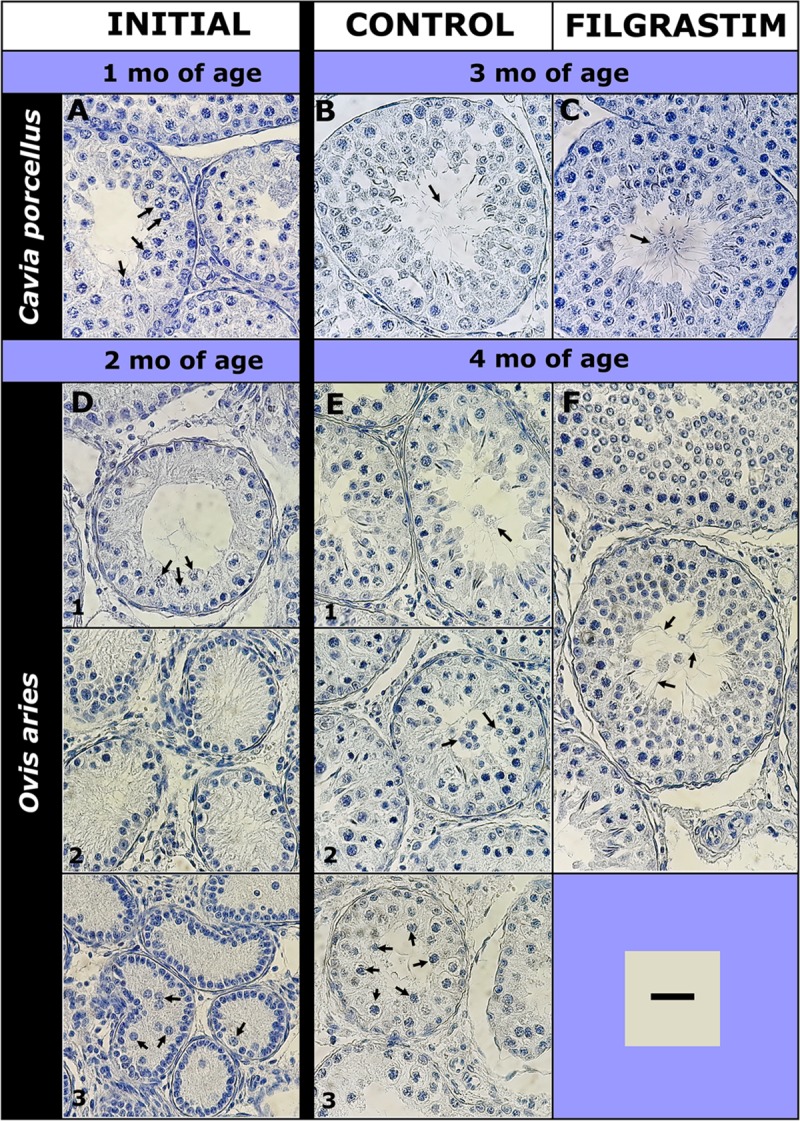
Seminifeorus tubule histology during r-met-hu G-CSF (Filgrastim) experiments considering the most advanced germ cell types. In guinea pigs (*Cavia porcellus*) experiments started at 1 mo of age and ended 2 mo after. Most advanced germ cell (GC) types at each time/treatment are shown with arrows: (A) spermatocytes; (B) and (C) sperm. In ram lambs (*Ovis aries*) experiments started at 2 mo of age and ended 2 mo after. At the beginning of the experiments, animals showed different phases of testicular development: (D1) spermatocytes; (D2) type A spermatogonia (D3) gonocytes. At the end of experiments there were control animals with (E1) sperm, (E2) round spermatids and (E3) sperm as the most advanced GC types and r-met-hu/G-CSF treated animals showing (F) sperm, as the most advanced GC type. GC types in the seminiferous epithelium were determined through histological/stereological analysis. *C*. *porcellus* initial n = 4; control n = 4, r-met-hu/G-CSF n = 4; *O*. *aries* initial n = 6; control n = 6, r-met-hu/G-CSF n = 5. Staining = hematoxylin. Bar = 30 μm.

In guinea pigs at day 0, 100% of the animals were already in spermiogenesis. After 60d, both control and r-met-hu/G-CSF treated guinea pigs (80 μg) were pubertal, with near 20% of them with incipient sperm production (less than 10% of seminiferous tubule cross-sections with sperm presence) and 80% with clear adult spermatogenesis, regardless of the treatment group (Table 3 and Fig 5). Pubertal guinea pigs from control and r-met-hu/G-CSF treated groups were compared in terms of daily sperm production (DSP) and spermatogenesis efficiency (DSP/g of testicular parenchyma). Guinea pigs DSP (p = 0.39) nor espermatogenic efficiency (p = 0.37) showed statistical differences between the control and r-met-hu/G-CSF treated group. Pooled DSP and DSP/g were 17.4 ±3.2 x 10^6^ and 11.9 ±2.8 x 10^6^, respectively.

**Table 3:** Summary of germ cell kinetics results during r-met-hu G-CSF experiments.

TESTICULAR DEVELOPMENT PHASES	Neonatal	Spermatogonial Expansion	Meiosis	Spermiogenesis	Puberty
**PREDOMINANT GERM CELL**	Gonocytes	Spermatogonia	Spermatocytes	Spermatids	Sperm
***Cavia porcellus***				Initial control (n = 4)	Control (n = 4) r-met-hu/G-CSF (n = 4)
***Ovis aries***	Initial control (n = 2)	Initial control (n = 2)	Control (n = 2) Initial control (n = 2)	Control (n = 2)	Control (n = 2) r-met-hu/G-CSF (n = 5)

Most advanced germ cell type status of animals in experimental groups at the time of castration is shown. “Initial control” are animals castrated at the beginning of the experiments (guinea pigs, *Cavia porcellus* age = 1 mo; ram lambs, *Ovis aries* age = 2 mo). r-met-hu/G-CSF animals received 20 μg/day (guinea pigs) or 360 μg/day (ram lambs) during 4 days and were castrated with their controls 2 months later. Animals (r-met-hu/G-CSF treated or controls) were assigned to a testicular development phase according to the most advanced cell types found in their seminiferous epithelium at the moment of castration, as determined through histological/stereological analysis. Numbers of animals are stated in the corresponding table cells.

No ram lamb testis before experiments was beyond meiosis: 50% of the animals were in neonatal stage (gonocytes as the most advanced cell type), 25% in spermatogenic stage and only 25% had entered meiosis. Thus, 75% of animals were on a pre-meiotic phase. After 60 days of experiments (r-met-hu/G-CSF treated with 1140 μg, vs. untreated controls), 33.33% of non treated animals had entered meiosis, 33.33% were in spermiogenesis and 33.33% were pubertal while r-met-hu/G-CSF treated animals were all pubertal (Table 3 and Fig 5). Ram lambs DSP (p = 0.26) nor espermatogenic efficiency (DSP/g, p = 0.38) showed statistical differences between the control and r-met-hu/G-CSF treated group. Pooled DSP and DSP/g were 79.6 ±28.4 x 10^6^ and 1.1 ±0.2 x 10^6^, respectively.

## Discussion

In this work we report for the first time the effects of r-met-hu G-CSF on sexual development by following haematological responses, testicular function, endocrine profiles and germ cell kinetics in prepubertal laboratory (*Cavia porcelus*) and farm (*Ovis aries*) animals to better understand the role of this growth factor in a non-hematopoietic tissue. Monitoring reproductive function and sexual development is crucial for the improvement of animal production programs [33–35]. Early puberty may play a critical role in domestic animal productivity and genetic/breeding programs [36,37]. However, there is scarce research on the use of exogenous stimulating factors to accelerate the onset of puberty. Filgrastim or r-met-hu G-CSF, a well characterized drug with effects on the physiology of hematopoiesis, has led to the development of a wide range of clinical life-saving applications [9,10].

The complex process of spermatogenesis starts for the first time during pre-pubertal development by the activation and proliferation of a pool of GCs. Gonocytes generate the first SSCs that will in turn be the foundation of adult spermatogenesis. Experimental efforts have previously hinted the *in vivo* role of G-CSF on spermatogenesis. Accordingly, GC depleting effects by cytotoxic drugs [19,20] or radiation [38] were reversed with G-CSF. We tested r-met-hu G-CSF during pre-puberty, a time window on which GCs expand and differentiate in a divergent way with intense germ stem cell activity. In a way, this is a natural system similar to a experimental GC depletion by cytotoxic agents and restoration through G-CSF. Our work provides evidence that G-CSF may be particularly involved in the first SSC proliferation wave at the start of spermatogenesis, at least in ram lambs.

r-met-hu G-CSF short term enhancement of WBCs mobilization on this work was as expected (in both studied species). Accordingly, peripheral WBCs increase in numbers within days of administration as reported in humans, pigs, horses, rats and mice [39–43]. The fact that guinea pigs WBCs, mainly granulocytes, peaked 2 days after administration with all doses levels used while in ram lambs all WBC types gradually increased in numbers right after r-met-hu G-CSF administration, indicates that r-met-hu/G-CSF doses were enough to activate and mobilize bone marrow hematopoietic stem cells in guinea pigs while in ovine species higher doses were required. In cancer therapy models differences on the pharmacodynamics of the drug have been observed. For instance, low doses of r-met-hu G-CSF are required in mice to recover hematopoiesis after irradiation [43] while pigs show hemopoietic mobilization after the use of relatively high doses (100 μg/Kg) [40]. Although r-met-hu/G-CSF shows high levels of biosafety [42], low doses (8 μg/Kg/day) are commonly used for therapeutic purposes [44].

Unchanged cortisol levels throughout experiments with ram lambs showed that the animals were under normal, steady stress conditions and also that the stress hormonal (adrenal) axis was kept intact under the effect of r-met-hu/G-CSF in this species (no differences between control and treated animals). However, there is evidence in the literature supporting both involvement [45] or not [46] of the adrenal axis after r-met-hu G-CSF administration, probably due to species-differences or experimental designs.

Even though blood cell dynamics was similar in guinea pigs and ram lambs treated with r-met-hu/G-CSF, reproductive hormone patterns differed significantly. Guinea pigs had one blood sample taken 5 days after r-met-hu G-CSF administration. In that specific time window, only LH was elevated in low r-met-hu/G-CSF -dosed animals while FSH and testosterone were unaltered, regardless of the dose. On the other hand, ram lambs had gonadotropin and testosterone values above those of control animals 5 days on after r-met-hu G-CSF administration. Overall effects of r-met-hu/G-CSF on guinea pig sexual development were not conclusive, perhaps due to species-specific sensitivity to the drug. Accordingly, with normal testosterone values found in r-met-hu/G-CSF treated guinea pigs, testis size and seminiferous tubule compartment were unchanged during the experiments. r-met-hu G-CSF treated guinea pigs did not show variations in the duration of the first spermatogenic wave leading to puberty since all animals were initially in spermiogenesis and the end of the experiments (after 2 mo) both groups (r-met-hu/G-CSF treated and control) were pubertal. Furthermore, in order to see the effect of r-met-hu G-CSF at the cell dynamics level, daily sperm production (DSP) and the efficiency of spermatogenesis (DSP/ g of testicular parenchyma) were estimated. Evaluation of spermatogenesis through these stereologic methods is based on key literature research through. Castration is often used and important/practical information derived in animal science [47]. For instance, testis biopsy evaluations are safely used to determine the reproductive status in rams [48]. No differences were found regarding these two parameters between r-met-hu/G-CSF treated and control groups suggesting that the first wave of spermatogenesis and early post-pubertal spermatogenic efficiency were not affected by r-met-hu G-CSF in guinea pigs.

r-met-hu/G-CSF effects on the sexual development of ram lambs showed a different pattern. Reproductive hormone progression in r-met-hu G-CSF treated animals is consistent with an activation of the Hypothalamus-Pituitary-Testis axis. Accordingly, higher testis weights were observed in treated animals. Moreover, r-met-hu/G-CSF treated animals were all pubertal at the end of the 2 mo experimental period while only about 25% of control animals were producing sperm. Interestingly, control animals were in the expected testis developmental age-range reported in the literature for several ovine breeds [49] but r-met-hu/G-CSF treated animals on this work had sperm earlier than have been reported. Consequently, G-CSF is involved in the regulatory mechanisms behind puberty, fertility and reproductive development [15,16,50,51]. The boost of gonadotropin levels after r-met-hu G-CSF administration we observed in ram lambs could hypothetically be traced back to the hypothalamus or the hypophysis gland. Although m-CSF is locally produced by astrocytes in the hypothalamus and might specifically signal GnRH-neurons (Cohen 1997) exogenous blood-borne m-CSF-like molecules would require crossing the blood-brain barrier (BBB) in order to induce GnRH-neurons to produce and secrete GnRH. As there is no evidence that G-CSF can come across the BBB the possibility remains that prepubertal animals might have an incompletely mature BBB at a time-window during development that r-met-hu/G-CSF could take advantage of to come across the barrier into GnRHergic nuclei in the hypothalamus. There are differences in rates of brain development among species and BBB development is not necessarily linked to the time of birth [52]. Although maturation of the BBB has been described in lambs as a continuous process from fetal to adult age (Stonestreet et al., 1996), details about the rates of barrier permeability changes, particularly during the prepubertal period, are not available. The possibility that r-met-hu/G-CSF could leak in through the barrier would explain, at least in part, the increased blood levels of gonadotropins found in the present study, probably via GnRH production and secretion. Besides the possibility of the BBB unstable permeability related to developmental maturation in different species, it has been reported that r-m/GM-CSF can cross BBB [53] as well as the blood-testis barrier (BTB) in mice [54]. Also in the mouse, other cytokines can cross the BTB such as Interleukin-1 alpha (IL-1 alpha) [55]. The molecular details of this passage through secluding biological barriers remains unknown and more studies are needed in other species. On the other hand, higher testosterone levels found in r-met-hu G-CSF treated ram lambs on this study probably took place via LH signaling (through previous hypothalamic-pituitary triggering), but also through local action in the testis as some research groups have shown. Thus, several cell types on the SSC niche in the testis produce m-CSF-1, such as Leydig cells, peritubular myoid cells and macrophages [50,56–58]. Furthermore, there is evidence CSF-1 is involved in steroidogenesis in Leydig cells. For instance, CSF-1^-/-^ mice show a general disregulation of the Hypothalamus-Pituitary-Testis axis that results in 90% lower testosterone concentrations compared to the wild type [59]. Furthermore, macrophages stimulate steroidogenesis in response to LH [60] and probably do so through physical contact with Leydig cells and cytokine secretion into their cytoplasm [61]. CSF-1 and biosimilars seem to control steroidogenic enzymes at a post-translational level, particularly P450_scc_, in the conversion of cholesterol to pregnelonone [62]. CSF-1 might also directly or indirectly feed back to the hypophysis and/or hypothalamus in yet to be described loops.

r-met-hu/G-CSF treated ram lambs, which had increased reproductive hormone values also had had heavier testes. Within the testis, CSF-Rs are located in SSCs [58,63,64]. CSF-1 stimulates SSC self-renewal in the testis (Oatley 2009) which may explain the effects of r-met-hu/G-CSF on testis weight and the apparently faster rate of the first wave of spermatogenesis in ram lambs. Seemingly, macrophages have a direct role on the SSC niche through CSF-1 and retinol pathways [65].

In the present work, r-met-hu G-CSF effect in extra-bone marrow tissues such as the male gonad seems to be long-term contrary to short-term hematopoietic effects. Species-specific effects were clearly observed. Guinea pigs long term reproductive unresponsiveness was probably due to non-equivalent developmental periods during the experiments compared to ram lambs, lower drug-sensibility, higher doses requirements or different cytokine (of human derived) -receptor affinities. Rams had their hypothalamus—pituitary–gonad axis activated and enhanced spermatogenesis with higher doses of r-met-hu G-CSF. More studies are needed in different species and under a wider range of r-met-hu G-CSF concentrations.

## Conclusions

In conclusion, the present work suggests r-met-hu G-CSF administration contributes to the unset of male mammalian puberty associated with highly species-specific responses during testicular development. Drug concentrations will require adjustments in order to modulate and improve the developmental kinetics of GCs with the aim of accelerating puberty arrival. Moreover, this study shows that the developmental effects derived from administering r-met-hu G-CSF in prepubertal male individuals leads to specific and differential endocrine responses and testicular developmental rates among in hystricomophs and ovine species. This supports the fact that species-specific protocols in the administration of r-met-hu G-CSF should be considered. r-met-hu G-CSF effects on sexual development were strongly evident in ovine species. With the due optimization, the use of r-met-hu G-CSF represents an efficient and functional method seeking to accelerate sexual developmental in mammalian species.

## Supporting information

S1 ARRIVE Guidelines checklist aponte. This file contains information on experimental animal welfare including methods to alleviate pain or suffering as well as animal monitoring schedules as presented in this manuscript.
(PDF)
